# Patient Partnership in Stroke Care: A Scoping Review

**DOI:** 10.7759/cureus.87446

**Published:** 2025-07-07

**Authors:** Hussain Bux, Furkan Zurel, Zakaria Rashid, Immanuel Sani

**Affiliations:** 1 Medical Education, Barking, Havering and Redbridge University Hospitals NHS Trust, London, GBR; 2 General Surgery, Salford Royal NHS Foundation Trust, Manchester, GBR; 3 Orthopaedics, Mid and South Essex NHS Foundation Trust, London, GBR; 4 Family Medicine, Leicester Medical School, Leicester, GBR

**Keywords:** communication, medical education, patient-centred care, patient partnership, stroke care

## Abstract

Patient partnership is increasingly recognised as a core component of high-quality healthcare. This review aims to explore how patient partnership is implemented across the stroke pathway, where recovery trajectories are complex and multidimensional. It will also show how medical education can shift student learning from a transactional process to one of partnership and collaboration.

A literature search was conducted across Cochrane Library, Embase, and PubMed, in accordance with the Preferred Reporting Items for Systematic Reviews and Meta-Analyses Extension for Scoping Reviews (PRISMA-ScR) guidelines. Studies were screened by title, abstract, and full text against eligibility criteria. Eleven articles were included in the review. Each article underwent quality appraisal using the Critical Appraisal Skills Programme (CASP) tool. Thematic synthesis was used, mapping findings onto three key stages of the Stroke RightCare Pathway: hyperacute, the stroke unit and early supported discharge, and long-term rehabilitation.

This review found that the role of the patient and clinician has changed in modern-day healthcare from paternalistic to one that aims to actively bring clinicians and patients together to form a collaborative partnership. Tailoring this partnership in each stage of stroke care is vital. In the hyperacute stroke setting, clinician-led decisions were found to be most beneficial for the patient due to the short time window to initiate treatment. During the acute phase, educating patients to understand their condition can allow for meaningful and collaborative goal-setting to take place, which has demonstrated improved patient outcomes. In medical education, introducing patient partnership early in a student’s training is essential for creating a lasting impact on healthcare.

Patient partnership must be tailored across the stroke pathway, and embedding these principles in medical education is important in shaping future clinicians' attitudes and practices. Future research should explore formal patient partnership training strategies for both students and clinicians. It should also assess whether clinicians find this approach appropriate across different medical conditions and clinical settings.

## Introduction and background

Patient partnership in healthcare is increasingly recognized as essential for improving patient outcomes and experiences. The King’s Fund defines patient partnership as a collaborative relationship where power is shared between clinicians and patients, moving beyond traditional hierarchical dynamics [1]. Patient experience, a key component of quality healthcare, is defined as ‘what the process of receiving care feels like for the patient, their family and carers’ [2]. Across global healthcare systems, the most effective healthcare experiences are shaped through strong patient-clinician relationships that emphasize respect, autonomy, continuity, and education [3].

Historically, healthcare models positioned clinicians as the sole decision-makers, reinforcing the misconception that only medical professionals hold the knowledge to guide treatment. However, since the 2014 NHS Five-Year Forward View, patient partnership has become the central focus of the NHS [4]. This shift represents a significant transformation in the role of patients in decision-making and care delivery. While many theoretical models of patient partnership have been developed, research on the practical application, particularly in improving patient experience, remains limited. This review will unpack the definition of patient partnership within the context of stroke care and explore how medical education, particularly at the undergraduate level, can transition from a transactional process to one that promotes partnership and shared decision-making with patients.

Why focus on stroke care?

Stroke is one of the leading causes of death and disability in the UK, with over 113,000 strokes occurring annually [5,6]. Although approximately 80% of stroke patients survive their hospital stay, many continue to experience long-term challenges [7,8]. With over 1.3 million stroke survivors in the UK, this number is expected to rise as the population ages and treatment methods develop [7,9]. Despite extensive research into the pharmacological and surgical management of stroke, the role of patient partnership in stroke recovery and long-term care remains underexplored.

Many stroke survivors report unmet long-term needs, particularly in activities of daily living (ADLs), home adaptations, and financial support [10]. A recent study found that nearly half of the stroke survivors in the UK had at least one unmet need following their stroke [10]. Addressing these gaps requires a patient partnership approach, ensuring survivors are actively engaged in their recovery and rehabilitation.

The Stroke Association recognises patient involvement as critical in improving stroke outcomes [11]. One of the top five research priorities in stroke care is delivering evidence-based treatment to improve survival and quality of life [10,12]. Studies suggest that patient engagement improves treatment adherence, functional outcomes, and overall satisfaction with care [13]. Given the increasing number of stroke survivors, integrating patient partnership within stroke care is not only beneficial but necessary to optimise recovery and long-term well-being.

The patient partnership model

**Figure 1:**
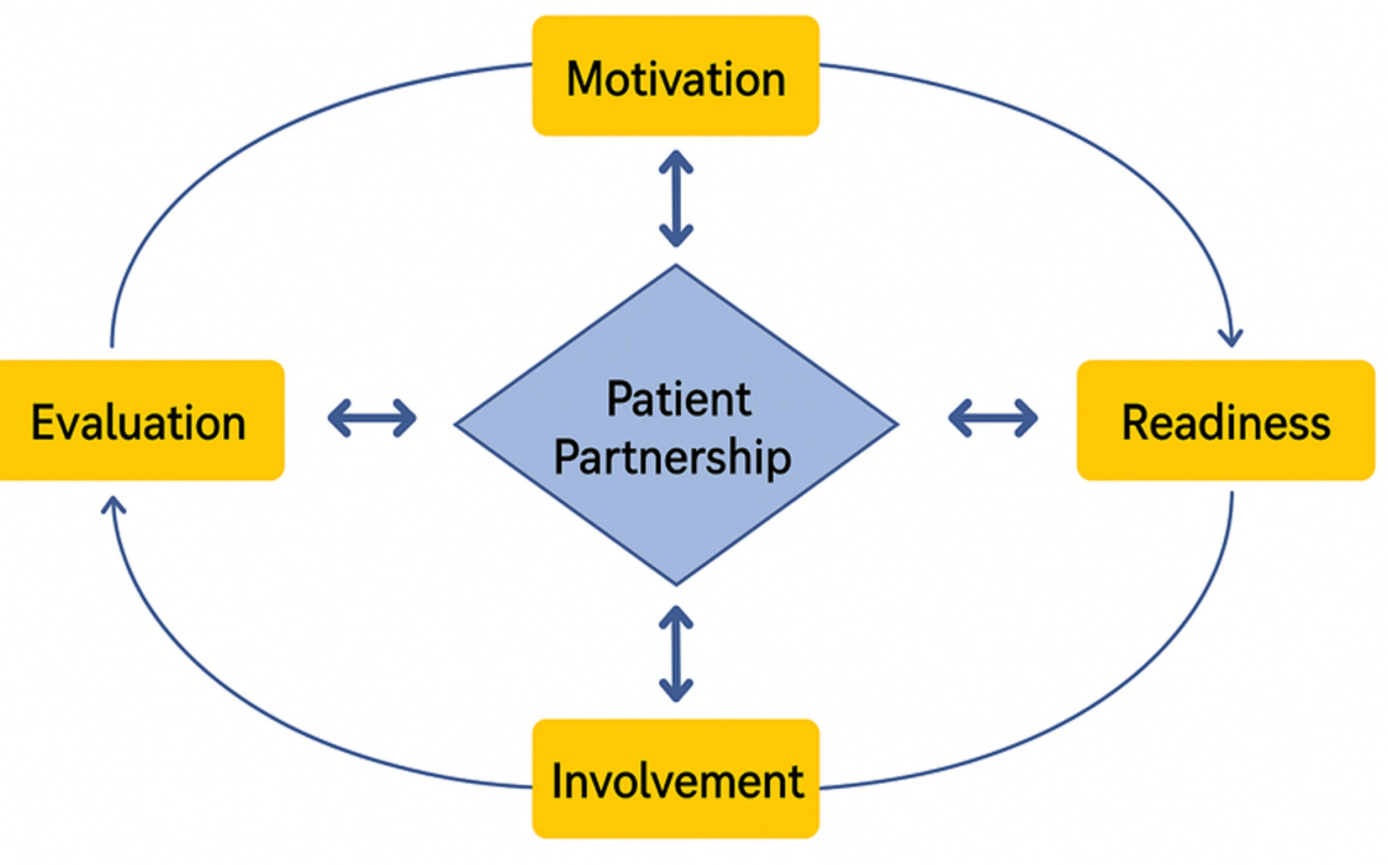
The patient partnership model. Figure credits: Hussain Bux. Source: Mohammadi et al. [14].

The patient partnership model has four stages: motivation, readiness, involvement, and evaluation [14], as summarised in Figure 1. Motivation involves the clinician and patient recognising the condition and the importance of active engagement [14]. Readiness focuses on preparing the patient with knowledge, allowing for active participation [14]. Involvement ensures continued collaboration to enhance health outcomes and quality of life [14]. Finally, regular evaluation assesses and strengthens the partnership for sustained and optimal outcomes [14].

Patient partnership: A life-long journey

**Figure 2:**
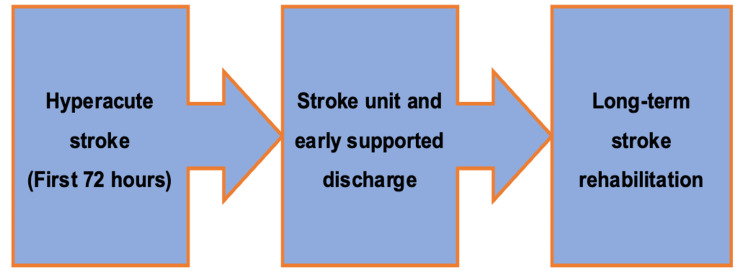
The Stroke RightCare Pathway. Figure credits: Hussain Bux. Source [15].

Collaboration between healthcare professionals and patients with stroke is a lifelong process. Understanding key interactions throughout the patient’s journey is essential for developing long-term engagement. This review will explore the role of patient partnership at each stage of the Stroke RightCare Pathway as summarised in Figure 2 [15]. This pathway is a national framework designed to improve stroke care in the UK through collaborative approaches [15]. By using the Stroke RightCare Pathway as the foundation of this review, we aim to demonstrate a holistic approach to integrating patient partnership and its impact on the stroke patient’s journey.

## Review

Methodology

**Figure 3:**
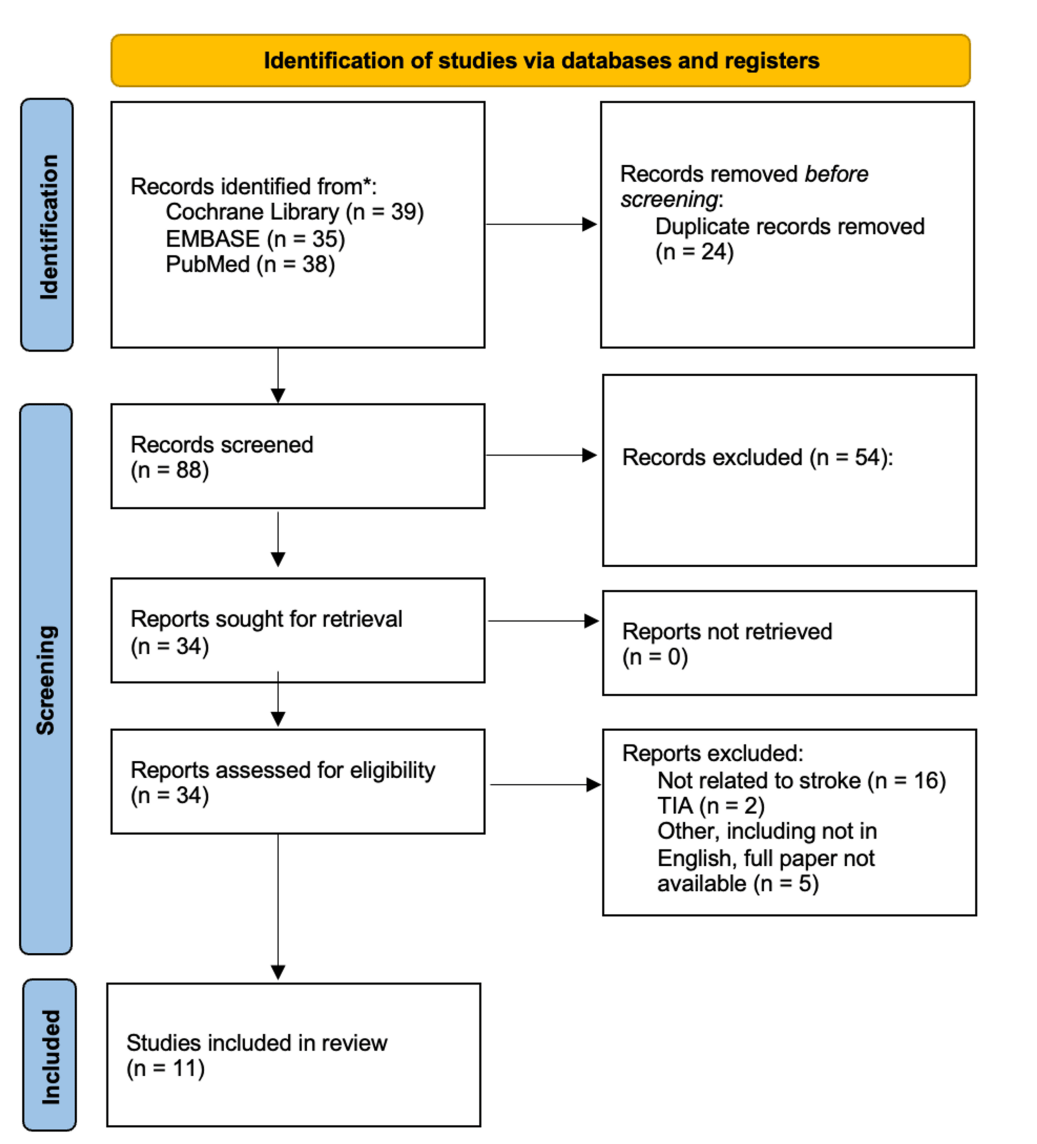
PRISMA flowchart. PRISMA: Preferred Reporting Items for Systematic Reviews and Meta-Analyses; TIA: transient ischemic attack. Figure created collaboratively by all four authors.

A comprehensive scoping review was performed following the Preferred Reporting Items for Systematic Reviews and Meta-Analyses (PRISMA) guidelines [16], as illustrated in Figure 3. The search was conducted using three databases: the Cochrane Library, Embase, and PubMed. The search strategy was designed to identify studies that explored patient partnership in stroke care, as well as the role of medical education in embedding these principles into clinical practice. The search terms are summarised in Table 1.

**Table 1:** Search items.

Core concept	Search terms
Patient partnership	Patient engagement, patient involvement, shared decision-making, patient-centred care, collaborative care, patient-clinician relationship.
Stroke care	Stroke, ischaemic stroke, haemorrhagic stroke, stroke recovery, stroke management, stroke rehabilitation, stroke outcomes.
Patient experience	Patient satisfaction, patient outcomes, patient perspective, patient education.
Medical education	Undergraduate medical education, medical training, medical school, teaching patient partnership.

The search strings used in the three databases combined Boolean operators (AND, OR) to ensure a broad yet relevant capture of studies: (1) Patient Partnership AND Stroke Care AND/OR Patient Experience; (2) Patient Partnership AND Medical Education AND/OR Stroke Care; (3) Patient Partnership AND Medical Education AND/OR Patient Experience.

Eligibility Criteria and Study Selection

Inclusion criteria: Adults aged 18 years or older with a history of stroke; studies exploring patient partnership in stroke management and recovery; patient involvement in undergraduate healthcare studies; both qualitative and quantitative research designs.

Exclusion criteria: Studies in languages other than English; unpublished literature; research not specifically examining patient-clinician collaboration or patient decision-making.

Data Extraction and Analysis

This review followed the Arksey and O’Malley framework for scoping reviews [17]. The scoping process began with one author (HB) reviewing titles and abstracts for eligibility. This was followed by an independent review by a second author (FZ) of the studies deemed potentially eligible. Following agreement on studies meeting the inclusion criteria between both authors (HB, FZ), the full texts of articles were assessed by both authors. Any discrepancies in study selection were resolved through discussion, with input from a third reviewer (IS) where necessary. Reference lists of included articles were manually searched by one author (HB) to identify additional papers potentially fulfilling the inclusion criteria.

The following data were collected from the included studies: study characteristics (e.g. authors, publication year, and study design); study participants; methodological approaches; key findings, including barriers and facilitators to patient partnership in stroke research, patient-related outcomes following stroke care, and institutional efforts to implement patient partnership in medical education.

The quality of each included study was assessed using the Critical Appraisal Skills Programme (CASP) tool [18], which evaluates three domains: (1) validity of results; (2) strength and clarity of findings; and (3) applicability to clinical or educational practice. A total score out of 10 was assigned to each study: high quality (≥8), moderate quality (5-7), and low quality (<5).

One author (HB) organised the extracted data into a summary table. To ensure accuracy, two additional authors (FZ, IS) independently extracted data from the included studies and compared their findings with HB’s. Any inconsistencies were resolved through discussion.

A thematic analysis was conducted to bring together the key findings from the included studies. An inductive approach was undertaken together by all four authors to identify patterns and themes emerging from the study findings. Subsequently, a deductive approach was undertaken by two authors (IS, ZR), using the NHS Stroke RightCare Pathway [15] framework to organise the findings into three overarching themes: (1) hyperacute stroke (first 72 hours); (2) stroke unit and early supported discharge; (3) long-term stroke rehabilitation.

This approach allowed for the initial exploration of new insights from the data without any preconceptions. Using both inductive and deductive methods ensured the thematic analysis was relevant to the study findings and clinically meaningful.

Results

Study Characteristics

Eleven studies were included in this review. The studies varied in sample sizes and methodologies, with participants including stroke patients, their families, healthcare professionals, and academic leads in medical education. A summary of key findings is presented in Table 2.

**Table 2:** Summary of included studies. FAST: face, arms, speech, time; MDT: multidisciplinary team; PCC: patient-centred care; CASP: Critical Appraisal Skills Programme.

Study	Study type	Number of participants	Key findings	Quality of paper using the CASP tool
Murtagh et al. (2012) [19]	Qualitative	62 participants (patients, families, healthcare professionals)	Challenges of patient partnership in hyperacute stroke setting: Lack of patient capacity, shock, time constraints. Patients and families valued clinician-led decision-making. Face-to-face communication was preferred over written material.	9/10. Well-conducted qualitative study, results clear, and highly relevant to patient partnership in acute care.
Harrison et al. (2013) [20]	Qualitative	59 participants (stroke survivors and carers)	Delays in treatment and communication issues were common. The "Act FAST" campaign improved pre-hospital delays, but patients and families faced dissatisfaction due to hospital delays. Communication from healthcare teams was often lacking, leading to anxiety and frustration in patients.	8/10. Well-conducted qualitative study, although it would benefit from useful findings for service improvement.
Bright et al. (2018) [21]	Qualitative	28 clinicians and 3 patients	Focused on the role of patient engagement in stroke patients with communication difficulties. Found that engagement is influenced by both patient and clinician partnership, with a shift from passive to active engagement.	7/10. Good qualitative study; however, a small number of participants, which can result in a lack of transferability to other settings, populations, or clinical contexts.
Simmons-Mackie & Damico (2009) [22]	Observational	6 participants	Highlighted the role of group therapy in patient engagement for stroke patients, and showed that peer conversations and MDT involvement could improve communicative strategies and patient satisfaction.	6/10. Useful insights, but small sample size limiting generalisability.
Rice et al. (2017) [23]	Qualitative	286 participants	Impairment-based goals were most common; patient involvement increased satisfaction.	8/10. Well-conducted qualitative study, results clear, and relevant to local practice.
Laver et al. (2010) [24]	Qualitative	15 participants	Many patients were unprepared to set goals early in recovery due to a lack of understanding.	7/10. Good qualitative study; however, a small number of participants, which can result in a lack of transferability.
Scobbie et al. (2015) [25]	Survey		91% of clinicians set goals with patients, but only 39% provided patients with copies of their goals.	7/10. Good data on clinicians; however, it lacks views on patient experiences. Useful for practice change.
Holliday et al. (2005) [26]	Quantitative		Patient-centred goal setting improved engagement and rehabilitation outcomes.	8/10. Well-conducted qualitative study, clear outcomes, and relevant to local practice.
Moore et al. (2021) [27]	Qualitative (interviews)	6 university leads	PCC included due to regulatory mandates, lacking clear implementation strategy.	7/10. Good for understanding educational context; however, not entirely applicable to local practice.
O’Keefe & Jones (2007) [28]	Qualitative (interviews)	1 medical school	Differences in attitudes towards patient involvement between faculty and patients.	6/10. Single medical school, limited data. Lacks wider applicability to local practice.
Jha et al. (2009) [29]	Mixed methods	46 participants	Faculty viewed patient involvement in assessments as tokenistic.	7/10. Good study and applicable to education.

Hyperacute Stroke: The First 72 Hours

Stroke patients are broadly classified into one of two main categories, ischaemic or haemorrhagic strokes, with ischaemic strokes accounting for 87% of cases [30,31]. An ischaemic stroke is caused by a sudden blockage of the blood vessel supplying blood flow to, or within the brain, often due to a thrombus formation or embolus [30,31]. In the hyperacute phase, impairments in communication and executive function can limit a patient’s ability to participate in shared decision-making [30,31].

Two key studies highlight the challenges of patient partnership in the hyperacute stroke setting [19,20]. Murtagh et al. (2012) conducted a qualitative study with 62 participants, including patients, families, and healthcare professionals, identifying major barriers to collaboration, such as patient incapacity, emotional shock, and time constraints [19]. The study found patients and families often preferred clinician-led decision-making rather than neutral choices, leaving the patient to decide [19]. Face-to-face communication was favoured over written materials; however, the small time window to apply thrombolysis coupled with the reduced availability of specialist stroke clinicians at any one time, restricted these interactions [19].

Similarly, Harrison et al. (2013) found that while the Act FAST campaign helped reduce pre-hospital delays, hospital experiences were often negative due to extended wait times and poor communication from staff [20]. Many patients, especially those with post-stroke communication difficulties, felt isolated and anxious due to limited clinician interaction [20]. The study highlighted the need for timely and clear communication to improve patient experience [20].

The Acute Stroke Unit and Early Supported Discharge

Early supported discharge (ESD) enables stable stroke patients to return home sooner, allowing rehabilitation and social support to continue in a familiar environment rather than an acute hospital ward [32,33]. Bright et al. (2018) conducted a qualitative study highlighting the importance of clinician-patient partnerships and demonstrated that active patient engagement depended on both the patient’s own efforts and the clinician’s communication approach [21]. This collaborative model improved patient satisfaction and rehabilitation outcomes [21].

Further research by Simmons-Mackie and Damico (2009) emphasised the role of the multidisciplinary team (MDT) and group therapy in enhancing communication and engagement [22]. The inclusion of professionals such as speech and language therapists helped overcome communication barriers, facilitated peer interactions, and improved patient satisfaction through the development of effective communicative strategies [22].

Long-Term Rehabilitation

Research on goal setting in long-term stroke rehabilitation highlights its benefits and challenges [34,35]. Rice et al. (2017) examined patient satisfaction with goal setting during outpatient rehabilitation, involving 286 stroke survivors [23]. The study found patients primarily set impairment-based goals aimed at improving body functions to enhance daily activities (ADLs) [23]. Greater patient involvement in goal setting was linked to higher satisfaction with rehabilitation, reinforcing its importance in recovery [23]. Similarly, Holliday et al. (2005) supported these findings, demonstrating that patient-centred goal setting positively influenced recovery experiences and engagement in rehabilitation [26].

Laver et al. (2010) explored stroke survivors' readiness to set goals at different recovery stages [24]. Semi-structured interviews were given to 15 stroke survivors aged between 18 and 70 years in three main settings: the acute stroke unit, a subacute rehabilitation programme, and six months after the stroke [24]. The study found that patients had difficulty setting goals early after a stroke, as nine of 15 patients felt they were not ready to set goals in the acute stroke unit due to a limited understanding of prognosis and goal-setting terminology [24]. Participants later recognised they had been unprepared and had little understanding of creating meaningful goals at the time, suggesting a need for structured education to support effective goal setting throughout rehabilitation [24].

Scobbie et al. (2015) investigated goal-setting practices from a healthcare professional perspective in community-based stroke rehabilitation [25]. A UK-wide clinician survey found that while goal setting was widely regarded as best practice, its implementation varied [25]. Although 91% of clinicians reported setting goals with most or all stroke patients, only 39% regularly provided patients with a copy of their goals, potentially limiting engagement [25]. This contrasts with stroke survivors' experiences in earlier studies, highlighting a gap between clinical practice and patient understanding of goal setting [25].

Implementing Patient Partnership in Medical Education

The studies reviewed highlight various approaches to integrating patient partnership within medical education, revealing both successes and challenges. While some institutions have made strides in embedding patient perspectives into training, inconsistencies in implementation and resistance from faculty remain key barriers.

Moore et al. (2021) investigated the representation of patient-centred care (PCC) in UK undergraduate healthcare programs [27]. Interviews with medical and nursing program leads revealed that PCC was often included due to regulatory requirements rather than a structured educational strategy [27]. A lack of consensus on its definition, implementation, and assessment contributed to inconsistent integration across institutions [27].

O’Keefe and Jones (2007) explored attitudes towards patient and community involvement in medical education, uncovering a divide between faculty staff and patients [28]. While patients advocated for broader community involvement, faculty members preferred collaboration with healthcare policymakers [28]. This highlights ongoing tensions regarding the role and influence of patient perspectives in curriculum design.

Despite these challenges, patient involvement in medical training continues to evolve in the UK. A 2018 BMJ report highlighted the inclusion of patients in assessments, such as Objective Structured Clinical Examinations (OSCEs), at institutions like King’s College London and the University of Manchester [36]. However, concerns persist regarding the reliability and objectivity of patient-awarded marks for medical students. Jha et al. (2009) echoed these concerns, by explaining that some faculty viewed patient participation in assessments as a tokenistic gesture rather than a meaningful contribution to the education of medical students [29].

Discussion

The studies reviewed highlight the significant challenges faced in the hyperacute stroke setting, where time constraints, limited patient capacity, and emotional distress complicate patient partnership [19,20]. Both Murtagh et al. (2012) and Harrison et al. (2013) emphasise the important role of clinicians in decision-making, particularly when patients and their families are unable to make informed choices due to shock or lack of capacity [19,20]. This dynamic contrasts with traditional concepts of patient partnership, which emphasise patient autonomy. In the hyperacute setting, the urgency for rapid intervention often takes precedence, and clinicians must balance immediate treatment needs with respect for patient autonomy. The ethical dilemma in hyperacute stroke care highlights a crucial gap in current practice: How can clinicians ensure truly informed consent when time is severely limited? While training in decision-making and communication is essential, systemic solutions, such as pre-established patient preferences or real-time digital consent tools, could mitigate the challenge of balancing immediate intervention with patient autonomy.

Effective communication remains a vital aspect of the patient experience. Both studies illustrated patients and families value face-to-face communication, which helps alleviate anxiety and promote trust in the treatment process [19,20]. When healthcare providers fail to communicate clearly and empathetically, it can negatively affect patient confidence and their overall experience [37]. Additionally, the lack of face-to-face communication can make patients feel isolated and anxious, particularly when they cannot communicate effectively after a stroke [37].

While improvements in organisational processes, such as better coordination with paramedics or pre-ordering CT scans, may reduce delays, they do not entirely address the need for clear and effective communication in the hyperacute setting [38,39]. A standardised communication protocol for hyperacute stroke settings could help bridge communication gaps. Keeping patients and their families informed about their situation, even if immediate action is not possible, can help maintain trust and confidence in their care [38,39]. While patient autonomy is limited in the hyperacute phase due to time constraints, the early rehabilitation period offers more opportunities for engagement. The following section explores how active patient involvement can improve recovery outcomes.

In the acute stroke unit and ESD process, engaging patients in their care during the early recovery phase is crucial for improving health outcomes and satisfaction [15,40,41]. Although patient engagement has been widely recognised as an important factor in various healthcare settings, its application in stroke care, particularly in ESD, remains under-researched. Across studies, it was found that active patient involvement leads to better rehabilitation outcomes, increased satisfaction, and a greater sense of control over recovery [42-44]. Bright et al. (2018) were among the first to emphasise the importance of clinician-patient collaboration, where effective engagement hinges on strong communication and patient participation [21]. Bright et al. (2018) highlight the need for clinicians to assess how they engage patients, particularly those with communication challenges, which are prevalent among stroke patients [21].

The biopsychosocial model is also crucial in guiding clinician-patient communication [21]. The use of the International Classification of Functioning, Disability, and Health (ICF) framework helps incorporate a holistic approach into clinical practice, considering the broader context of patients’ lives, abilities, and social roles [45].

A wide range of socially determined factors can significantly impact stroke patients’ ability to engage in their care [45]. These include socioeconomic status, level of education, employment, housing stability, access to transportation, cultural and linguistic background, and family or social support networks [45]. Patients with limited financial resources may struggle to afford ongoing therapy, adaptive equipment, or transportation to appointments, all of which can reduce access to consistent, high-quality rehabilitation. Employment status may also affect access to healthcare benefits or time availability for attending sessions. Patients who live alone or are without carers may face greater difficulties adhering to rehabilitation programs or accessing emotional support. Conversely, strong support networks can improve motivation and engagement, particularly for individuals with communication or cognitive impairments [21,45]. Therefore, clinicians must be aware of and responsive to these diverse social determinants to deliver patient-centred stroke care.

**Figure 4:**
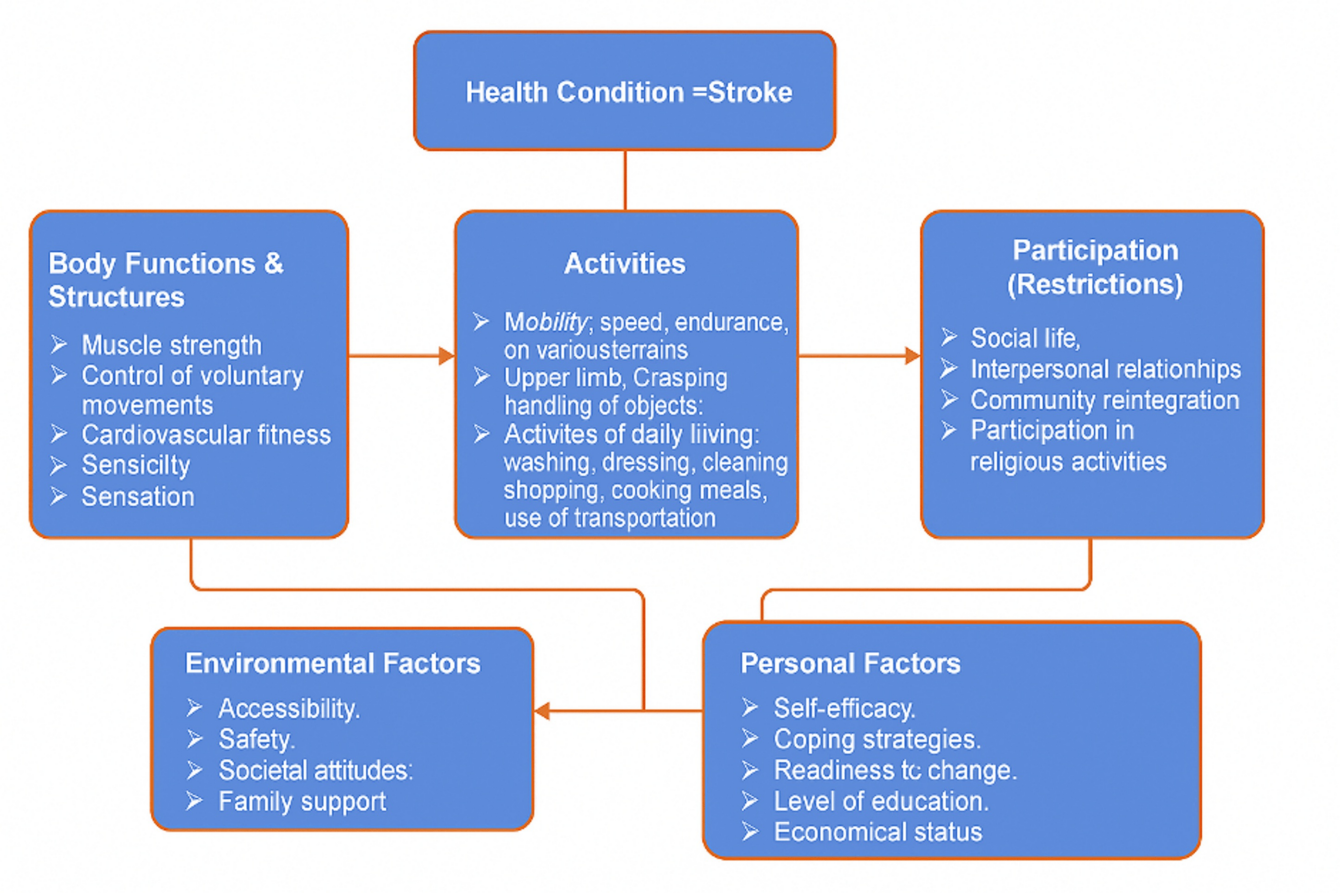
The International Classification of Functioning, Disability and Health (ICF) framework for stroke. Figure credits: Hussain Bux and Immanuel Sani. Source: Kostanjsek [45].

The use of the ICF framework should be the foundation of a clinician's management [21,45]. The ICF framework can provide a holistic view of the patient’s functions, activities, and social roles within a broader context [45]. A summary of this model is summarised in Figure 4. The framework can allow the patient to be directly involved alongside the clinician in their management journey [21,45]. However, it is important to point out that patients may not be aware of the role they can play in patient engagement. Being unaware of what patient engagement is and the role patients can play can result in patients often reducing themselves to listening to what the practitioner is telling them rather than actively participating in discussions and taking control of their condition engagement. This can result in a dictatorship between the clinician and patient rather than a partnership, resulting in disengagement and potentially a negative impact on long-term health outcomes. To tackle this, efforts must be made to not only raise patient awareness of the partnership role they can play in their management journey but also, as suggested by Bright et al. (2018), make changes from an organisational level to include stroke patients in policy conversations, positions of responsibility, and leadership [21,45]. These two changes can result in local, regional, and potentially national implementation of patient engagement and patient partnership rather than single cases of patient engagement in isolation. Thus, if the two changes are implemented successfully with traction, it can result in patient partnership becoming an occurrence in every health visit rather than a research phenomenon, which is rarely seen in healthcare.

However, despite these advances in theory and understanding, many stroke patients still face barriers to engagement, particularly those with aphasia or other communication difficulties [46]. Bright et al. (2018) examined the role of communication in patient engagement, emphasizing clinicians can involve patients with significant communication difficulties in decision-making with appropriate support [21]. This aligns with previous literature, demonstrating that patients who feel engaged in care decisions report better outcomes and greater satisfaction [43,44,47,48]. Overcoming communication barriers, particularly for patients with aphasia or cognitive impairments, requires tailored strategies involving the MDT to facilitate participation. Speech and language therapists (SALT) play a crucial role in addressing communication challenges, including expressive language and swallowing difficulties.

Simmons-Mackie and Damico (2009) suggest collaborating with different members of the MDT improves patient engagement by offering a personalised approach to communication, addressing each patient’s unique needs [22]. Strategies like communication boards, speech-generating devices, and visual aids can improve comprehension and support patient participation in their own care [22]. Additionally, providing information in short, simple statements and allowing ample time for patients to process and respond can improve effective communication further [22]. By integrating MDT professionals, healthcare teams can mitigate communication barriers and ensure patients are more engaged in their care process, developing trust and a sense of inclusion [22].

Despite these promising findings, challenges remain in translating these insights into clinical practice. A recurring theme in the studies was the impact of communication difficulties on patient engagement, especially among patients with aphasia or cognitive impairments [21,22]. These challenges can lead to patients being passive recipients of care, reducing satisfaction and slowing their recovery process [21,22]. Clinicians face additional barriers such as time constraints, lack of resources, and a lack of training in communication strategies [22]. To overcome these barriers, mandatory training modules on patient engagement, including adaptive communication strategies and shared decision-making, should be integrated into the training curriculum. Raising awareness about patient engagement and providing practical tools for clinicians can help improve the patient experience and health outcomes. It is particularly important to equip patients with the knowledge and skills needed to actively participate in their care.

The studies on long-term rehabilitation emphasise the complexities of goal setting, highlighting both its benefits and barriers. A recurring theme is the importance of patient involvement in setting goals. Rice et al. (2017) demonstrated that increased participation in goal setting correlated with higher satisfaction [23]. However, as Laver et al. (2010) identified, many patients struggle with goal setting in the early stages of recovery due to readiness in setting goals, as well as a limited understanding of stroke recovery [24]. This suggests while goal setting is a valuable tool, its timing and delivery need careful consideration to maximise positive patient health outcomes.

Scobbie et al. (2015) found that while clinicians widely acknowledge the importance of goal setting, many fail to provide patients with copies of their goals [25]. Laver et al. (2010) found stroke survivors expressed difficulties understanding the goal-setting process, especially early in recovery [24]. These discrepancies highlight the need for a more structured patient partnership approach, ensuring that stroke survivors are well-informed and actively involved in rehabilitation planning.

Education plays a key role in bridging this gap. Laver et al. (2010) and Holliday et al. (2005) suggested stroke survivors benefit from clear information about their condition, the rehabilitation process, and the role of goal setting in their recovery [24,26]. Without adequate education, patients may struggle to set realistic goals, leading to disengagement and frustration. Integrating structured education with goal-setting within the patient partnership model could empower patients in their recovery journey [14]. Despite goal setting being emphasised in clinical guidelines, these studies demonstrate more work is needed to optimise its implementation.

Patient Partnership in Medical Education

The studies reviewed [27-29] demonstrate the potential and ongoing challenges of integrating patient partnership in medical education. While institutions have taken steps to embed patient perspectives, these efforts are often driven by external regulatory requirements, which can result in a box-ticking exercise. This, in turn, can form an outcome where patient perspectives may not be meaningfully embedded within the curriculum, limiting their impact on shaping future healthcare professionals’ understanding and responsiveness to patient needs.

Moore et al. (2021) found program leads agreed the main reason for the inclusion of PCC in undergraduate healthcare programmes was due to the requirements set out by the General Medical Council (GMC) and the Nursing and Midwifery Council (NMC), which are independent regulators in the UK for doctors and nurses and oversee their respective teaching qualifications in higher education [27]. Moore et al. (2021) demonstrated a common theme among the higher education programme leads was that there was no clear clarity on the definition of PCC, how to implement PCC within the curriculum, and how to assess the competence of healthcare students to ensure they have the competence to apply PCC in practice [27]. This mirrors broader difficulties in patient partnership, where institutions may support the concept in theory but struggle with its practical application. O’Keefe and Jones (2007) further illustrated this divide, noting patients advocated for more community involvement, while faculty members emphasised academic and regulatory influences [28].

Despite these challenges, there is emerging evidence that patient involvement enhances student learning. The University of Manchester’s Doubleday Centre has pioneered direct patient involvement in teaching, while King’s College London has incorporated patient-assessed OSCEs. However, concerns raised by faculty, such as those outlined by Jha et al. (2009), demonstrated more rigorous methods for training and evaluating patient assessors may be necessary to ensure fairness and reliability in assessments [29].

Moving forward, the next steps should focus on establishing a structured, widely accepted framework for patient partnership in medical education. This includes clearer definitions, methods for assessment, and ongoing faculty development to ensure patient involvement is meaningful rather than symbolic. Addressing these gaps will help move medical education towards a more holistic and sustainable model of patient partnership, benefiting both students and the healthcare system.

Limitations

There are a number of limitations in this review. Firstly, this review was limited to studies in the English language only. This may have excluded relevant findings from non-English speaking countries where cultural attitudes towards patient partnership may differ. Secondly, the term ‘patient partnership’ lacks a consistent definition across studies, which may have affected the interpretation of some of the studies. Thirdly, there were a small number of studies included.

Another key limitation of this review is the reliance on qualitative, retrospective interviews in many of the studies reviewed. For example, some of the studies were based on self-reported experiences, which may be influenced by bias. Self-reported experiences introduce subjectivity and potential recall bias, particularly as stroke survivors may overemphasise negative experiences due to emotional distress. This limitation raises concerns about the reliability of findings and suggests a need for real-time data collection methods, such as patient diaries or wearable technology, that can capture experiences more objectively.

While this review provides valuable insights into stroke care, its narrow focus on a single condition limits its applicability to other conditions. Nevertheless, the insights gained from this review contribute meaningfully to the understanding of patient partnership in stroke care and reinforce the importance of the patient's voice in clinical and educational practice.

Future research

Moving forward, for academics, as the patient partnership continues to gain traction, more research on the four key pillars of the patient partnership, i.e., motivation, readiness, involvement, and evaluation, in different medical conditions needs to be considered to see if similar outcomes are found. Research should also focus on practical methods for overcoming the barriers to patient partnership, ensuring it becomes a standard practice that enhances both patient care and medical education. Beyond clinician training, future research should explore technology-driven solutions for patient partnership. AI-powered speech devices, mobile applications for real-time patient updates, and virtual reality (VR) training modules could revolutionise engagement strategies in stroke care [49].

## Conclusions

This scoping review highlights the challenges and opportunities involved in promoting patient partnership in both stroke care and medical education. This review provides an understanding of patient partnership, where clinicians and patients can break down the metaphorical wall of a division and work towards a collaborative relationship where power is shared. This review highlights that patient partnership improves adherence, better health outcomes, and a more positive patient experience. This review found that in a hyperacute setting, where time is of the essence and patients and family members may still be in shock, the clinician’s expertise and clinician-led decisions were found to be most beneficial for the patient. During the acute phase of a stroke, actively engaging patients in their management plan has been proven to improve patient satisfaction and health outcomes. Moreover, educating patients to understand the condition and what a typical recovery from a stroke looks like can allow for meaningful and collaborative goal-setting to take place, which can, in turn, improve patient satisfaction. This review highlights that patient partnership improves adherence, better health outcomes, and a more positive patient experience. By embracing patient partnership in both healthcare delivery and education, we can create a healthcare system that is truly responsive to the needs of the patients it serves.

## References

[1] Seale B (2016). The King's Fund. Patients as partners. https://assets.kingsfund.org.uk/f/256914/x/13533b47b3/patients_as_partnersjuly_2016.pdf.

[2] NHS Institute for Innovation and Improvement (2024). The Patient Experience Book. https://www.england.nhs.uk/improvement-hub/wp-content/uploads/sites/44/2017/11/Patient-Experience-Guidance-and-Support.pdf.

[3] Vahdat S, Hamzehgardeshi L, Hessam S, Hamzehgardeshi Z (2014). Patient involvement in health care decision making: a review. Iran Red Crescent Med J.

[4] Maruthappu M, Sood HS, Keogh B (2014). The NHS five year forward view: transforming care. Br J Gen Pract.

[5] ISD Scotland (2017). Scottish National Audit Programme (SNAP). Scottish Stroke Improvement Programme Report-2016. Scottish Stroke Care Audit, Scottish Stroke Improvement Programme Report-2016.

[6] (2017). Sentinel Stroke National Audit Programme (SSNAP). http://bit.ly/1NHYlqH.

[7] (2024). Stroke statistics. https://www.stroke.org.uk/stroke/statistics#UK%20summary.

[8] OECD OECD (2021). Health at a Glance 2021: OECD Indicators.

[9] King D, Wittenberg R, Patel A, Quayyum Z, Berdunov V, Knapp M (2020). The future incidence, prevalence and costs of stroke in the UK. Age Ageing.

[10] (2022). Stroke priority setting partnership. https://www.stroke.org.uk/research/priority-setting-partnership.

[11] Patel A, Berdunov V, King D, Quayyum Z, Wittenberg R, Knapp M (2022). Current, Future and Avoidable Costs of Stroke in the UK. Executive Summary Part 2: Societal Costs of Stroke in the Next 20 Years and Potential Returns From Increased Spending on Research. Stroke Association.

[12] Sumsion T, Law M (2006). A review of evidence on the conceptual elements informing client-centred practice. Can J Occup Ther.

[13] Jones F, Riazi A (2011). Self-efficacy and self-management after stroke: a systematic review. Disabil Rehabil.

[14] Mohammadi E, Abedi HA, Jalali F, Gofranipour F, Kazemnejad A (2006). Evaluation of 'partnership care model' in the control of hypertension. Int J Nurs Pract.

[15] (2022). RightCare Pathway: Stroke. https://www.england.nhs.uk/rightcare/wp-content/uploads/sites/40/2017/09/stroke-pathway.pdf.

[16] Tricco AC, Lillie E, Zarin W (2018). PRISMA Extension for Scoping Reviews (PRISMA-ScR): checklist and explanation. Ann Intern Med.

[17] Arksey H, O'Malley L (2005). Scoping studies: towards a methodological framework. Int J Soc Res Methodol.

[18] (2025). Critical Appraisal Skills Programme (CASP) checklists. https://casp-uk.net/.

[19] Murtagh MJ, Burges Watson DL, Jenkings KN, Lie ML, Mackintosh JE, Ford GA, Thomson RG (2012). Situationally-sensitive knowledge translation and relational decision making in hyperacute stroke: a qualitative study. PLoS One.

[20] Harrison M, Ryan T, Gardiner C, Jones A (2013). Patients' and carers' experiences of gaining access to acute stroke care: a qualitative study. Emerg Med J.

[21] Bright FA, Kayes NM, McPherson KM, Worrall LE (2018). Engaging people experiencing communication disability in stroke rehabilitation: a qualitative study. Int J Lang Commun Disord.

[22] Simmons-Mackie N, Damico JS (2009). Engagement in group therapy for aphasia. Semin Speech Lang.

[23] Rice DB, McIntyre A, Mirkowski M, Janzen S, Viana R, Britt E, Teasell R (2017). Patient-centered goal setting in a hospital-based outpatient stroke rehabilitation center. PM R.

[24] Laver K, Halbert J, Stewart M, Crotty M (2010). Patient readiness and ability to set recovery goals during the first 6 months after stroke. J Allied Health.

[25] Scobbie L, Duncan EA, Brady MC, Wyke S (2015). Goal setting practice in services delivering community-based stroke rehabilitation: a United Kingdom (UK) wide survey. Disabil Rehabil.

[26] Holliday RC, Antoun M, Playford ED (2005). A survey of goal-setting methods used in rehabilitation. Neurorehabil Neural Repair.

[27] Moore HL, Farnworth A, Watson R, Giles K, Tomson D, Thomson RG (2021). Inclusion of person-centred care in medical and nursing undergraduate curricula in the UK: interviews and documentary analysis. Patient Educ Couns.

[28] O'Keefe M, Jones A (2007). Promoting lay participation in medical school curriculum development: lay and faculty perceptions. Med Educ.

[29] Jha V, Quinton ND, Bekker HL, Roberts TE (2009). What educators and students really think about using patients as teachers in medical education: a qualitative study. Med Educ.

[30] Yousufuddin M, Young N (2019). Aging and ischemic stroke. Aging (Albany NY).

[31] Yaghi S, Willey JZ, Cucchiara B (2017). Treatment and outcome of hemorrhagic transformation after intravenous alteplase in acute ischemic stroke: a scientific statement for healthcare professionals from the American Heart Association/American Stroke Association. Stroke.

[32] Melnychuk M, Morris S, Black G (2019). Variation in quality of acute stroke care by day and time of admission: prospective cohort study of weekday and weekend centralised hyperacute stroke unit care and non-centralised services. BMJ Open.

[33] (2024). Department of Health. National Stroke Strategy. https://nsnf.org.uk/assets/documents/dh_081059.pdf.

[34] McKevitt C, Fudge N, Redfern J (2011). Self-reported long-term needs after stroke. Stroke.

[35] Sumathipala K, Radcliffe E, Sadler E, Wolfe CD, McKevitt C (2012). Identifying the long-term needs of stroke survivors using the International Classification of Functioning, Disability and Health. Chronic Illn.

[36] Wilkinson E (2018). The patients who decide what makes a good doctor. BMJ.

[37] (2022). Patient-centered care: improving quality and safety through partnership with patients and consumers. https://www.safetyandquality.gov.au/sites/default/files/migrated/PCC_Paper_August.pdf.

[38] Kendall J, Dutta D, Brown E (2015). Reducing delay to stroke thrombolysis—lessons learnt from the Stroke 90 Project. Emerg Med J.

[39] Meretoja A, Strbian D, Mustanoja S, Tatlisumak T, Lindsberg PJ, Kaste M (2012). Reducing in-hospital delay to 20 minutes in stroke thrombolysis. Neurology.

[40] Langhorne P, Baylan S (2017). Early supported discharge services for people with acute stroke. Cochrane Database Syst Rev.

[41] Hebert D, Lindsay MP, McIntyre A (2016). Canadian stroke best practice recommendations: stroke rehabilitation practice guidelines, update 2015. Int J Stroke.

[42] Laws D, Amato S (2010). Incorporating bedside reporting into change-of-shift report. Rehabil Nurs.

[43] Kortte KB, Falk LD, Castillo RC, Johnson-Greene D, Wegener ST (2007). The Hopkins Rehabilitation Engagement Rating Scale: development and psychometric properties. Arch Phys Med Rehabil.

[44] Lequerica AH, Kortte K (2010). Therapeutic engagement: a proposed model of engagement in medical rehabilitation. Am J Phys Med Rehabil.

[45] Kostanjsek N (2011). Use of the International Classification of Functioning, Disability and Health (ICF) as a conceptual framework and common language for disability statistics and health information systems. BMC Public Health.

[46] O'Halloran R, Worrall L, Hickson L (2011). Environmental factors that influence communication between patients and their healthcare providers in acute hospital stroke units: an observational study. Int J Lang Commun Disord.

[47] Miles A, Watt T, Wong WY, McHutchison L, Friary P (2016). Complex feeding decisions: perceptions of staff, patients, and their families in the inpatient hospital setting. Gerontol Geriatr Med.

[48] Hemsley B, Balandin S (2014). A metasynthesis of patient-provider communication in hospital for patients with severe communication disabilities: informing new translational research. Augment Altern Commun.

[49] (2024). OpenAI. ChatGPT. https://openai.com.

